# Interactions of Medial and Lateral Prefrontal Cortex in Hierarchical Predictive Coding

**DOI:** 10.3389/fncom.2021.605271

**Published:** 2021-02-03

**Authors:** William H. Alexander, Thilo Womelsdorf

**Affiliations:** ^1^Center for Complex Systems and Brain Sciences, Florida Atlantic University, Boca Raton, FL, United States; ^2^Department of Psychology, Vanderbilt University, Nashville, TN, United States

**Keywords:** medial prefrontal cortex, lateral prefrontal cortex, cognitive control, attention, learning, predictive coding, computational neuroscience

## Abstract

Cognitive control and decision-making rely on the interplay of medial and lateral prefrontal cortex (mPFC/lPFC), particularly for circumstances in which correct behavior requires integrating and selecting among multiple sources of interrelated information. While the interaction between mPFC and lPFC is generally acknowledged as a crucial circuit in adaptive behavior, the nature of this interaction remains open to debate, with various proposals suggesting complementary roles in (i) signaling the need for and implementing control, (ii) identifying and selecting appropriate behavioral policies from a candidate set, and (iii) constructing behavioral schemata for performance of structured tasks. Although these proposed roles capture salient aspects of conjoint mPFC/lPFC function, none are sufficiently well-specified to provide a detailed account of the continuous interaction of the two regions during ongoing behavior. A recent computational model of mPFC and lPFC, the Hierarchical Error Representation (HER) model, places the regions within the framework of hierarchical predictive coding, and suggests how they interact during behavioral periods preceding and following salient events. In this manuscript, we extend the HER model to incorporate real-time temporal dynamics and demonstrate how the extended model is able to capture single-unit neurophysiological, behavioral, and network effects previously reported in the literature. Our results add to the wide range of results that can be accounted for by the HER model, and provide further evidence for predictive coding as a unifying framework for understanding PFC function and organization.

## Introduction

Medial and lateral prefrontal cortex (mPFC/lPFC) are core hubs of the cognitive control and decision-making network in the brain (Cole and Schneider, 2007). The regions are densely and reciprocally connected (Barbas and Pandya, 1989; Barbas and Rempel-Clower, 1997; Nácher et al., 2019), suggesting that their contribution to behavior depends in part on their tightly-coupled interactions during preparation, execution, and monitoring of the consequences of actions. Although these regions have long been the target of focused investigation, it remains an open question as to how they collaborate in an ongoing and interactive fashion to support adaptive behavior (Badre and Nee, 2018).

Independently, both regions have been implicated in a wide range of functions, and these functions appear to suggest a dissociation between the regions along a temporal dimension. Activity in mPFC, especially dorsal anterior cingulate cortex (dACC), is frequently observed in conjunction with behaviorally-salient events, such as error commission (Gehring et al., 1990; Shen et al., 2015) or the appearance of stimuli cueing multiple, potentially conflicting, responses (Brown, 2009). In contrast, activity in lPFC is typically associated with long-term behavioral contingencies, such as maintaining information over extended periods of time (Sawaguchi and Goldman-Rakic, 1991) and representing the structure of ongoing tasks (Badre and D'Esposito, 2007). This temporal dissociation has been interpreted as reflecting complementary aspects of task performance in which mPFC signals changes in behavioral requirements, including specification of control signals (Shenhav et al., 2013) or selection of action policies (Holroyd and Yeung, 2012), and the implementation of appropriate control measures is delegated to lPFC (Botvinick et al., 2001).

**Figure 1:**
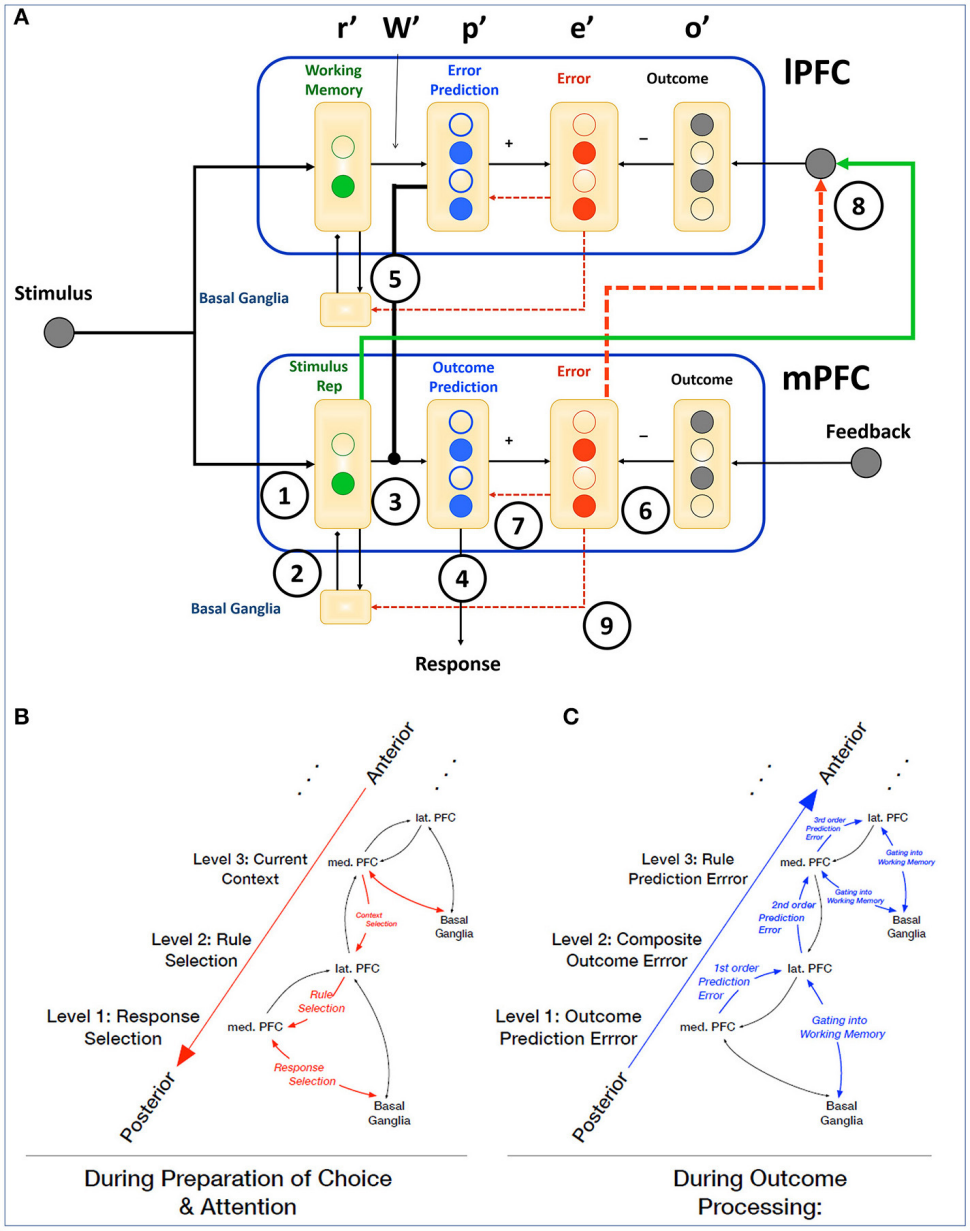
Interactions of mPFC and lPFC in the HER model. **(A)** The HER model is organized hierarchically, with each hierarchical level instantiating a computational motif of prediction and prediction error calculations. Information flows between hierarchical levels along top-down and bottom-up pathways, which carry information regarding likely errors in a given context (top-down), and error signals (bottom-up) derived from external feedback (base hierarchical level) conjoined with items maintained in working memory (additional hierarchical levels). Circled numbers indicate governing equations within and between hierarchical levels (see section Methods). Reciprocal connections between mPFC and lPFC correspond to a putative hierarchical rostrocaudal gradient (Badre and D'Esposito, 2009) with bottom-up and top-down pathways governing the adjustment of behavior during ongoing task performance. **(B)** During a trial, top-down connections serve to establish the current context and relevant rules that govern eventual behavioral responses during preparatory periods. **(C)** Following the generation of a response (right), bottom-up error signals are used to update outcome predictions and derive composite “proxy” outcomes that train higher-order representations of rules and contexts.

How such temporally specific functional contributions of medial and lateral PFC can give rise to a wide variety of cognitive and behavioral effects has been formalized within the framework of hierarchical predictive coding (Alexander and Brown, 2015, 2018). The Hierarchical Error Representation (HER) model states that error signals generated by mPFC are used to train *error representations* in lPFC, and that, once learned, error representations maintained by lPFC serve to contextualize subsequent error calculations carried out by mPFC (Figure 1). Using this basic circuit as a repeating computational motif, the HER model is able to learn complex cognitive tasks in a manner that accords with human behavior, and measures of activity derived from error calculation and representation in the model reproduce qualitative patterns of single-unit and neuroimaging data.

While the HER model suggests how and when mPFC and lPFC might interact, our previous implementation of the model was aimed at the “event” level (Alexander and Brown, 2015): model equations were applied, and activity derived, following the occurrence of salient behavioral events, such as the presentation of task stimuli or performance feedback. The HER model is therefore only able to account for mPFC/lPFC interactions at a temporally coarse level, and is unable to capture the co-evolving development of activity during intra-event periods, nor can it capture aspects of behavior, such as switch costs (Wylie and Allport, 2000), that may manifest through differences in response time. Thus, although the HER model provides a promising framework for understanding the function, organization and interactions of PFC regions (Alexander and Brown, 2018), a significant gap in its explanatory power remains.

To address this limitation and derive novel model predictions, we here incorporate temporal dynamics to the model-unit activity of the HER model. When model units transform inputs to outputs during performing of a reversal learning task we simulate their activation with a non-linear “shunting” equation (Grossberg, 1988). These dynamics reproduce the rise and fall of neuronal activations in cortical circuits. This allowed us to analyze the timing and duration of computations at different hierarchical levels of the model and to relate these dynamics to the behavioral, single-unit, and brain network data of empirical studies.

## Methods

### Model Equations

Details regarding the operation of the HER model are described in previous publications (Alexander and Brown, 2015). Here we review the equations specifying the model, adopting the notational convention that **BOLD UPPERCASE** variables indicate matrices, **bold lower case** variables indicate column vectors, and *lowercase italicized* variables indicate scalars. Variables marked with a single apostrophe (e.g., **P'**) indicate superior hierarchical levels. Table 1 lists parameters in the model, their interpretation, and their value for the simulations reported here. Table 2 lists model variables, their interpretation, and equations in which they appear.

**Table 1:** Model Parameters.

			**Parameter Values**	
**Parameter**	**Description**	**Equation**	**Hierarchical Level 1**	**Hierarchical Level 2**
α	Response learning rate	7	0.05	0.02
λ	Working memory learning rate	10	0.3	0.5
β	Working memory update gain	2	12	14
γ	Response selection temperature	4	12	N/A

**Table 2:** Model Variables.

**Variable**	**Description**	**Equation**
**v**	Utility for storing item in WM	(1, 2)
**X**	Associative WM weights	(2, 10)
**s**	Stimulus vector	(1, 10)
**p**	Model-generated predictions	(3, 4, 6)
**P**	Matricized predictions (**p**)	(5)
**W**	Associated response weights	(3, 5, 7, 9)
**r**	Working memory representation	(3, 7, 8, 9)
**e**	Error	(6, 7, 8)
**o**	Observed response-outcome vector	(6, 8)
**e_WM_**	Backpropagated working memory error	(9, 10)

Generally, the HER model is composed of 2 or more hierarchical levels, and each level of the hierarchy consists of three primary components: a working memory (WM) store ***r***, and weight matrix **X** determining the probability that a stimulus **s** will be stored in **r**, and a weight matrix **W** determining how items stored in WM either influence behavior (at the lowest hierarchical level) or modulate the processing of lower hierarchical levels. Whether a stimulus **s** is stored in working memory is determined by the pair of equations:

(1) $$\begin{array}{l} \text{v}={\text{X}}^{\text{T}}\text{s} \end{array}$$

(2) $$\begin{array}{l} \text{and the probability of WM representation }{\text{r}}_{\text{i}}\;=\;\frac{\exp^{\text{β}v_{i}}}{\exp^{\text{β}v_{i}}+\exp^{\text{β}v_{j}}} \end{array}$$

Equation (1) reflects the learned value of storing an item in working memory—at each hierarchical level of the model. Here, **s** is a binary vector of stimulus representations reflecting the current trial, with each element indicating the presence (1) or absence (0) of the corresponding task feature (cf. the task description below). The relative values of maintaining the current contents of WM (indexed by *j*) vs. updating WM with a new representation (indexed by *i*) are compared in Equation (2). Equation (2) is a softmax function calculating the probability of storing the current stimulus *s*_*i*_ in WM relative to the current contents of WM and β is a gain parameter governing the degree is a gain parameter governing the degree by which differences in elements of ***v***influence the probability of maintaining or updating WM. Intuitively, these equations determine which of multiple “valuable” to store in WM (Equation 1), and transform this value into a probability (Equation 2). WM memory transform this value into a probability (Equation 2). WM memory storage is binary, and only a single WM any time.

The WM representation **r** and associative weights **W** are used to generate the output **p** of that hierarchical level:

(3) $$\begin{array}{l} \text{p }=\;{\text{W}}^{\text{T}}\text{r} \end{array}$$

At the lowest hierarchical level, network output **p** is used to generate a behavioral response *i* (out of j possible responses) using another softmax function over elements of **p**:

(4) $$\begin{array}{l} \text{Probability of response }i=\frac{exp^{\gamma p_{i}}}{\sum_{j}exp^{\gamma p_{j}}} \end{array}$$

where γ is a gain parameter indicating how likely the model is to select the response *i* from *j* possible responses. The output of higher-order hierarchical levels, **p'**, is used to modulate the processing of lower-order levels output as follows. First, **p'** is reshaped from a vector to a matrix with the same dimensionality as **W**. That is, **p'** is matricized as **P'**. Next, modulated weight matrices are calculated as:

(5) $$\begin{array}{l} W^{mod}=W+P^{\prime } \end{array}$$

Note that **p'** is constrained to have the same number of elements as **W** so that **P'** has the same dimensionality following matricization.

### Learning

Learning in the model depends on the calculation of error signals at each level of hierarchy. At the base hierarchical level, errors are computed as:

(6) $$\begin{array}{l} \text{e }=\text{ a }\circ \;\left(\text{o}-\text{p}\right) \end{array}$$

where **o** is a feedback vector indicating the response identity and whether the response was correct or incorrect, and **a** is a filter that is 1 for the index of the generated response and 0 everywhere else. The operator **°** reflects elementwise multiplication (Hadamard product) of two matrices. Associative weights at each level are updated according to:

(7) $$\begin{array}{l} {\text{W}}_{\text{t}+1}={\text{W}}_{\text{t}}+\text{ α}\left({\text{e}}_{\text{t}}{{\text{r}}_{\text{t}}}^{\text{T}}\right) \end{array}$$

and α is the learning rate. To train higher-order hierarchical levels, a proxy outcome signal is composited from the error signal computed at the lower level (Equation 6) and the active WM representation **r** at the lower level. This is done by taking the outer product of the lower-level error and representation vectors:

(8) $$\begin{array}{l} {\text{O}}^{\text{′}}=\text{r}{\text{e}}^{\text{T}} \end{array}$$

For computational convenience, **O'** is vectorized as **o'** with the same dimensionality as **p'**.

Training of WM weights **X** is done by backpropagating the output error term **e** to derive an error term for the current WM representation:

(9) $$\begin{array}{l} {\text{e}}_{\text{WM}}\;={\text{W}}_{t}^{\text{T}}{\text{e}}_{t}\;∙\;{\text{r}}_{t} \end{array}$$

(10) $$\begin{array}{l} {\text{X}}_{t+1}={\text{X}}_{t}+\lambda \left({\text{e}}_{\text{WM }}{\text{s}}_{t}^{\text{T}}\right) \end{array}$$

with learning rate λ.

### Simulated Task

**Figure 2:**
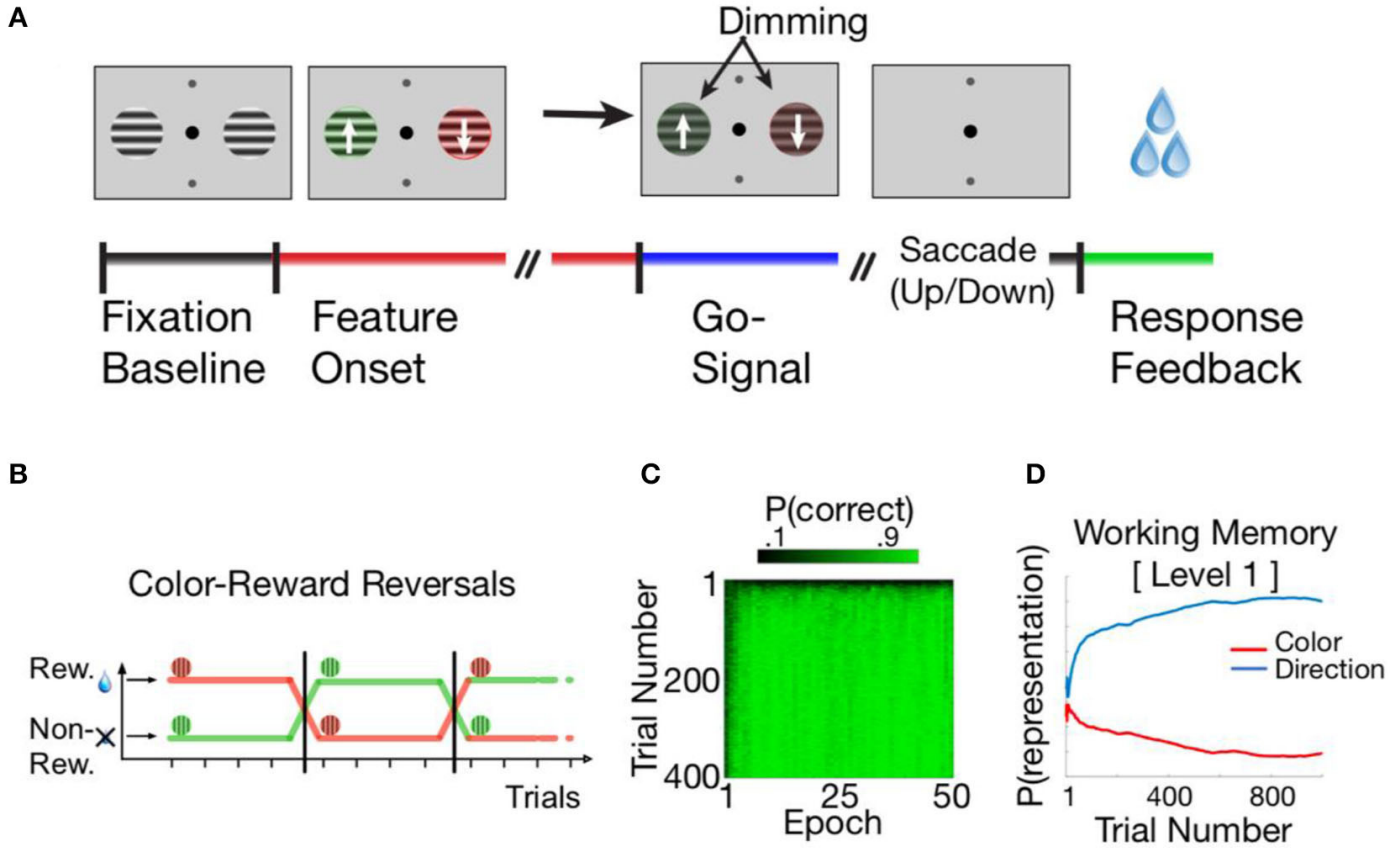
**(A)** The color-based reversal task described in Oemisch et al. (2019). Subjects are shown a fixation point and two neutral stimuli. Then the stimuli switched to opposite colors and began to move within their apertures in opposite, upward, or downward directions of motion. Following a brief interval, a “Go” (dimming) signal is presented, following which the subject is required to indicate the direction of the grid for the currently relevant color. The dimming signals occurred at unpredictable moments in time either in both stimuli simultaneously or in sequence to control covert attention (not shown). **(B)** The rewarded, relevant color reversed uncued after ≥30 trials, and subjects had to adjust their behavior to indicate the movement direction associated with the newly relevant color. **(C)** The HER model is able to learn the Oemisch et al. reversal task easily. During an initial period lasting from 1 to 10 reversal epochs, the model learns to preferentially gate in task features to hierarchical levels. Following reversals, the model rapidly learns the new task contingencies while preserving the hierarchical order of information. **(D)** The concrete decision variable in the reversal task is the apparent direction of motion associated with the currently relevant color. The HER model learns to represent this variable at the lowest hierarchical level (Level 1), consistent with its direct relevance to generating behavioral responses.

The HER model was developed to account for behavior and brain function in tasks requiring the selection and application of rules that govern how to respond to a concrete stimulus (e.g., an arrow cueing response identity). We therefore selected a recent, rule-based task (Oemisch et al., 2019) in which, on each trial, monkeys were presented two stimuli, one on each side of the screen (Figure 2A). Stimuli had two behaviorally relevant features that varied independently from each other, namely color (red/green), and the direction of motion of a grating inside a circular aperture (up/down). The task required that the monkey learn through trial-and-error which of the two colors is currently relevant, and to respond with a saccade in the up- or downward motion direction of the stimulus with the currently relevant color. Once the relevant color has been learned, successful performance of the task requires that, on each trial, the monkey first identifies the ***location***(side of the screen) of the appropriately-colored stimulus, after which the identity of the ***response***(up/down) corresponding to the direction of the pattern movement is determined. The task described in Oemisch et al. additionally incorporates periodic reversals when the relevant color changes and the monkey must adapt their responses to the new task contingencies (Figure 2B); while the HER model is able to learn such reversals (cf. Figure 2C), this aspect of the task is not further considered here as it does not bear directly on the *within-trial* temporal dynamics of PFC interactions.

### Model Simulations

To simulate real-time dynamics in the HER model, we adopt the approach taken by previous studies of network models of cognitive control [e.g., Yeung et al. (2004)] in which the process used to establish weights suitable for performing a task is carried out independently of simulations of temporal dynamics. In order to establish model weights suitable for performing the Oemisch et al. reversal task, a previously-described version of the (event-level) HER model (Alexander and Brown, 2015) using the same parameters was trained on 20,000 trials of the reversal task, divided into 50 blocks of 400 trials each. On each trial during learning, 3 events were modeled: the onset of task stimuli, the occurrence of a response and feedback, and a neutral cue indicating the start of the intertrial interval (ITI). Stimuli were modeled as a binary vector, with elements corresponding to color and movement direction (four total elements per stimulus), and independent stimulus vectors were used to model each side of the simulated display, for a total of eight task stimulus elements. A 9th element was used to indicate the ITI. The model was permitted 3 responses, two corresponding to movement direction (up/down), and one neutral response indicating acknowledgment of ITI onset. Each response could be associated with two outcomes (correct/incorrect), for a total of 6 response-outcome predictions. Responses were generated by subtracting, for each response, the prediction of incorrect feedback from the prediction of correct feedback, and passing the values through a softmax function, as described in the methods section. Code for the HER model is available at https://www.github.com/modelbrains.

### Temporal Dynamics

The network weights recovered from the training procedure were fixed during the real-time simulations as described above. In order to simulate real-time dynamics in the network, changes in unit activity on each cycle were modeled by a non-linear “shunting” equation (Grossberg, 1988):

(11) $$\begin{array}{l} \Delta E_{i}=exc_{i}\left(\theta -E_{i}\right)-\left(E_{i}+\eta \right)+N\left(0,\sigma \right) \end{array}$$

where θ is the upper asymptotic activity a unit could achieve (set to a value of 10), η is the lower boundary toward which unit activity decays passively (−0.05), and *N* is gaussian noise applied to the signal change with mean 0 and variance σ = 0.01. *E*_*i*_ is the current activity of unit *i*, and *exc*_*i*_ is the current net excitatory input to the unit, computed as in equations 1, 3, and 6. Specifically, exc_i_ is equal to **v** in Equation (1), governing the dynamics of WM update and maintenance. For computing model predictions at all hierarchical hierarchical levels, *exc*_*i*_ is equal to **p** in Equation (3). Finally, for computing error (Equation 6), *exc*_*i*_ is set to **e**, the difference between predictions **p** and observed outcomes **o**.

Models of neuron activity similar to Equation (11) [e.g., Wilson and Cowan (1972)] approximate the dynamics of individual neurons (Hodgkin, 1969) without introducing unnecessary complexity. The approach to simulate activity dynamics with a typical neuronal activation function has advantages over the use of biophysical model neurons when models are complex and the aim is to understand the algorithmic as opposed to the implementational level of a computational problem (Niv and Langdon, 2016). The HER model architecture has not previously been implemented into a biophysical model in order to allow for an analysis of the model mechanisms at the level of the computational units across hierarchy as opposed to the level of biophysical mechanisms underlying these units.

Each simulated trial lasted up to 700 cycles, and on each cycle the processing steps outlined in Equations (1–10) were followed and unit activity updated according to Equation (11). During the first 100 cycles of the trial, no input was presented to the network. On cycle 101, a stimulus vector representing the color and movement direction of the left and right stimuli was presented to the network, after which the network was able to register a response. Responses were generated when the corresponding unit in **p** reached 95% of its upper asymptotic value. Following a response, the stimulus vector was set to 0 and the network was provided feedback for 50 cycles. Following the offset of feedback, the network was run for an additional 250 cycles prior to the beginning of the next trial. Network activity was not re-initialized after each trial. As described in previous work (Alexander and Brown, 2011, 2014, 2015, 2018), Equations (1–10) specify two primary signal types, namely prediction (Equations 1 and 3) and error (Equation 6), and analyses were conducted on unit activity reflecting individual network units recruited during each trial. In the simulations reported reported here, both types of signals are subject to the temporal dynamics embodied in Equation (11). All model results were derived from 20 simulation runs.

### Granger Causality

We recorded the time course of unit activity for prediction (Equation 3) and error (Equation 6) signals from both hierarchical levels of the model. To determine the average causality between regions for each type of signal, we used the Granger Causality Toolbox (Luo et al., 2013; https://www.dcs.warwick.ac.uk/~feng/causality.html).The goal of Granger causality is to determine whether the value of a signal **y** at time *t* is better predicted by the history of another signal, **x**, than by its own history. The magnitude of Granger causality is given by the log ratio of the error when considering only the history of **y** vs. the history of both **y** and **x**.When the history of **x** does not improve predictions of **y** at time *t* above only the history of **y**, the log ratioi sequal to 0 (i.e., the error is the same in both cases).

The length of the signal history to be considered is determined by the analyst; here we selected a time window of 50 model cycles. For causality analyses, trial timing was standardized—even if a response was indicated prior, the model was simulated for 350 cycles following the onset of a stimulus. The average Granger causality magnitude of one hierarchical level on the other for each signal type was estimated for each cycle. This procedure was repeated 20 times, and a two-sample *t*-test (Bonferroni corrected) was conducted for the distribution of Granger causality magnitudes at each cycle to determine whether the granger causality of one region on the other was significantly greater. Thus, although both regions could Granger-cause activity in the other at any time point, our analysis indicated whether one region had a significantly higher degree of causality than the other.

## Results

The HER model was able to learn and perform the reversal task relatively easily. During an initial “burn-in” period (Figure 2C), the model primarily learned to hierarchically segregate relevant feature dimensions (Figure 2D): the WM “gating” mechanism (Equations 1 and 2) learned to store the “concrete” movement direction at the lowest hierarchical level while color information was stored in the superior hierarchical level. This pattern accords with intuitions regarding how individuals might solve the task (Oemisch et al., 2019): The reward-associated color acts as a feature-reward “rule” that indicates which concrete stimulus (movement direction) should govern the ultimate response. After this initial learning period, the model is able to perform reversals within a short period of time. Because the model has learned stable WM mappings, learning to respond to the opposite color entails relatively rapid changes in top-down modulation of concrete responses, i.e., rather than relearning the task from the ground up. These findings suggest one manner in which a hierarchical representation of information might support rapid and flexible reconfiguration of responses in the face of changing task contingencies.

### Response Preparation

**Figure 3:**
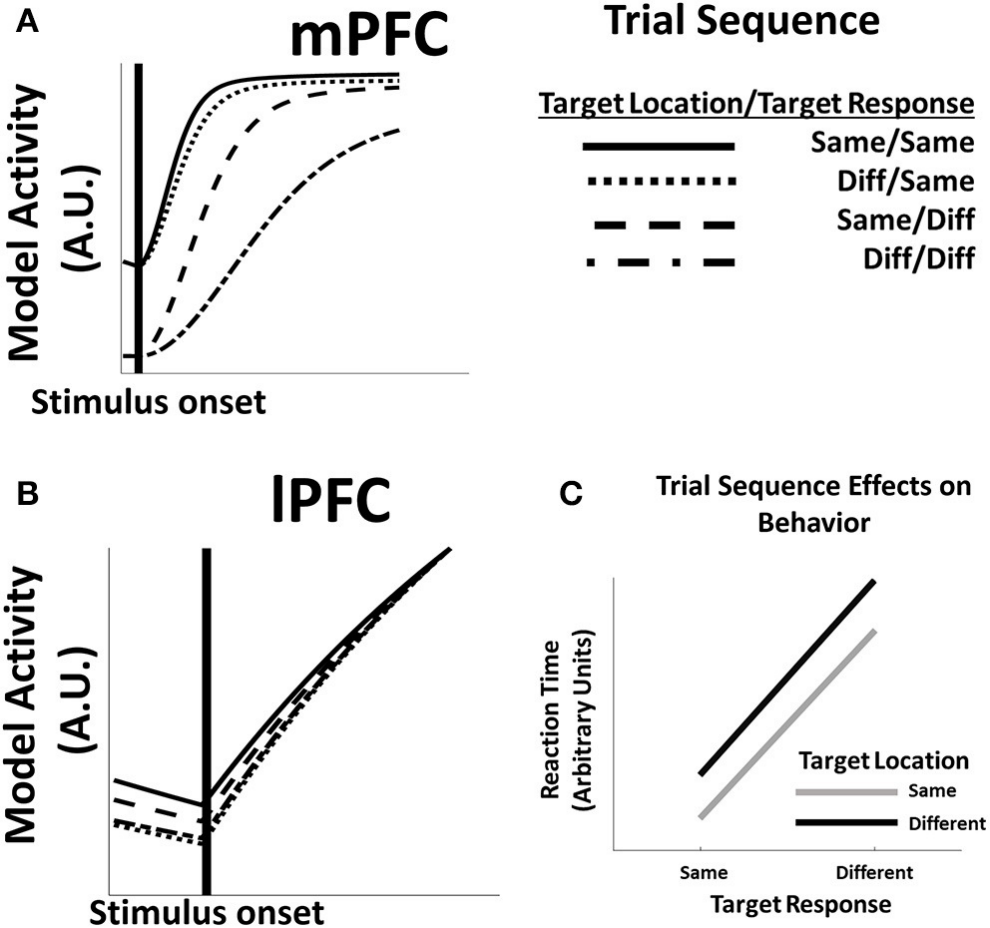
**(A)** mPFC unit activity in model simulations is influenced by switches or repetitions of feature dimensions between trials. When both feature dimensions (location and response direction) repeat (**same/same**), as well as repetitions of the target direction (**diff/same**), mPFC activity reaches asymptote quickly after stimulus onset, while changes in the target response (**same/diff, diff/diff**) produce delays in the evolution of mPFC activity. **(B)** Activity in lPFC likewise shows intertrial effects; however, in this case, delay in the development of lPFC activity is due principally to shifts in the location of the target stimulus (**diff/same, diff/diff**), while changes in the more concrete target direction variable (**same/diff**) have relatively little influence on lPFC activity relative to a trial in which all features are the same (**same/same**). **(C)** The delay in model activity following feature switches contributes to changes in model reaction times: reaction times are most rapid on trials in which both feature dimensions repeat, while switches in either or both features result in longer reaction times.

Introducing real-time dynamics allows us to investigate the evolution of predictive activity (Equation 3) in the model during a trial, the influence of previous trial effects, and derive measures of reaction time from model activity. During preparatory periods following the onset of a task stimulus the activity of predictive units in both mPFC (Figure 3A) and lPFC (Figure 3B) begins to ramp up, and the time course of this ramping up depends on whether task features in the current trial are the same as or different than features in the preceding trial. In lPFC, activity increases more rapidly for trials in which the target location is the same as the previous trial. This rapid increase is a consequence of lPFC's role in representing color information needed to identify the location of the target stimulus during a trial: when the location remains the same, lingering activity in the appropriate lPFC representations are rapidly re-activated. In contrast, mPFC activity is sensitive to changes both in the location of the target stimulus, as well as changes in the cued direction; both factors influence the development of mPFC activity. The sensitivity of mPFC in changes to both stimulus features is due in part to lingering activity within MPFC from the previous trial. As with the effect of location repetitions on lPFC activity, repetitions in the motion direction of the target stimulus re-activate mPFC representations that were already partially active from the previous trial. However, activity in mPFC is additionally modulated by activity in lPFC (Equation 5); changes in the location of the target stimulus that influence the evolution of lPFC activity thus have downstream effects on mPFC activity.

The sequence-dependent development of activity directly influences the speed at which a response is generated (Figure 3C). Responses in the model are ultimately generated by mPFC function (Equation 4): the longer activity in mPFC takes to reach a critical threshold, the slower the response time. Because the evolution of mPFC activity in the model depends on whether task features for the current trial are the same, the model reproduces standard trial sequence effects wherein feature repetition facilitates responding, while feature switches interfere with responses (Fecteau and Munoz, 2003). The HER model further suggests why some feature switches may produce greater interference effects than others. Specifically, switches of the feature that most directly drives response, i.e., the motion direction of the stimulus, results in a greater RT difference than changes in the more abstract feature dimension, i.e., the color of the stimulus in this task.

**Figure 4:**
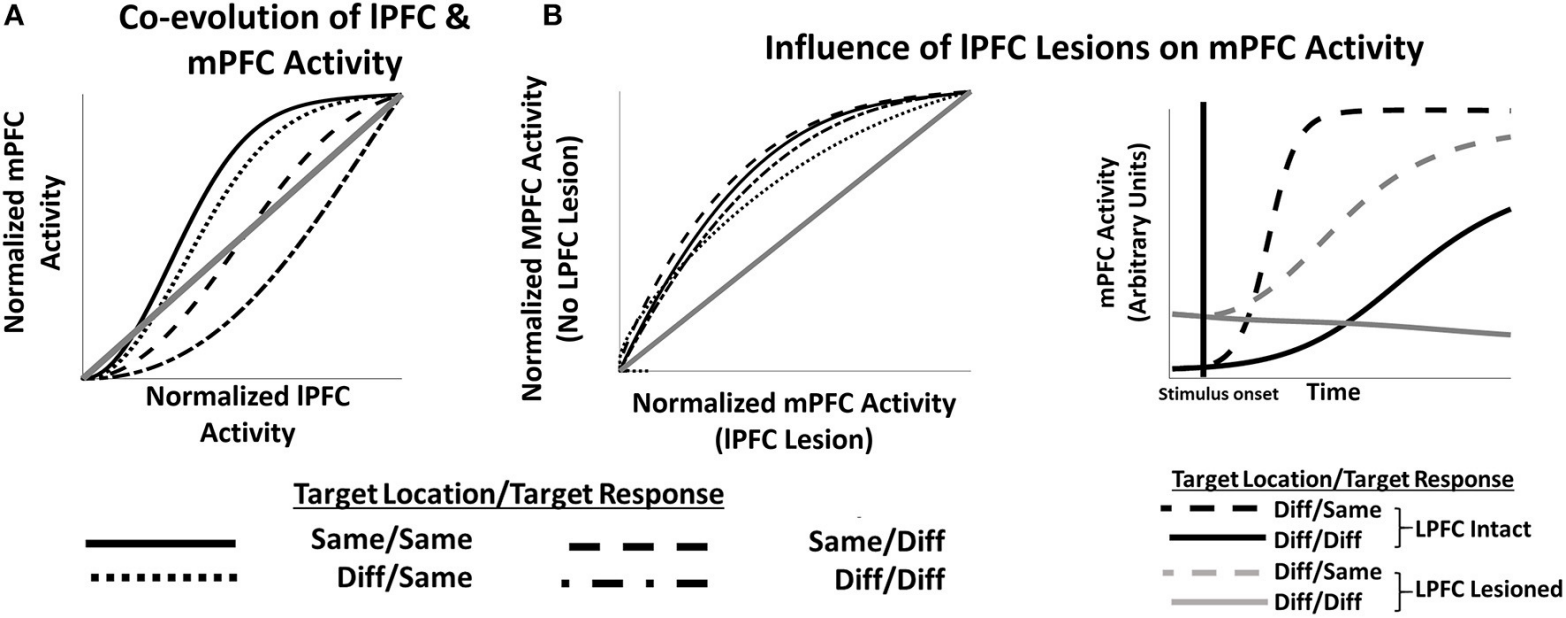
**(A)** lPFC activity develops more quickly than mPFC activity during early trial stages, consistent with a causal role for lPFC in modulating mPFC responses. **(B)** Left frame. Artificially lesioning the simulated lPFC component of the model results in slower development of mPFC activity (*x* axis) compared to a non-lesioned version of the model (*y* axis) irrespective of trial sequences condition. However (Right Frame), mPFC function is more severely impacted by lPFC lesions in some conditions than others.

Furthermore, the model suggests how lPFC lesions might affect the development of mPFC activity (and consequent behavioral performance) on tasks that require integrating multiple features (Figure 4). The evolution of both LFPC and mPFC activity is affected by whether the abstract task variable (location) in the current trial is the same or different than in the previous trial (Figure 4A), and lPFC activity tends to lead mPFC activity, suggesting a causal role of lPFC activity on mPFC activity. This causal relationship can be directly queried by artificially lesioning lPFC in the network (e.g., by multiplying **P'** by zero in Equation 5). mPFC activity following lesions to lPFC (Figure 4B, left frame) is slower to evolve than when lPFC is intact. When considering only trials in which the location of the target stimulus has changed (Figure 4B, right frame), lPFC lesions more profoundly impact mPFC function when both target location and target response identity have changed relative to changes only in the target location.

### Feedback Processing

Following a response, the model receives feedback indicating whether the selected response was correct or incorrect. While equations used to compute errors in the model (Equation 6), like those used to calculate prediction unit activity, apply at every moment in the simulations, error-related activity is most prominent following feedback delivery, during which ongoing predictive activity is compared to an experienced outcome. During learning in the event-level model, comparison of feedback and concrete outcomes occurs only at the lowest hierarchical level; at superior hierarchical levels, “outcomes” are derived from WM representations at the inferior level combined with the results of the feedback comparison process (i.e., the error signal, Equation 8). These “proxy” outcomes constitute a higher-order training signal that is composited from lower-level WM representations and error signals; the composition of the higher-order training signal is carried out by lPFC in the HER model, while the comparison of outcomes and predictions is undertaken by mPFC.

**Figure 5:**
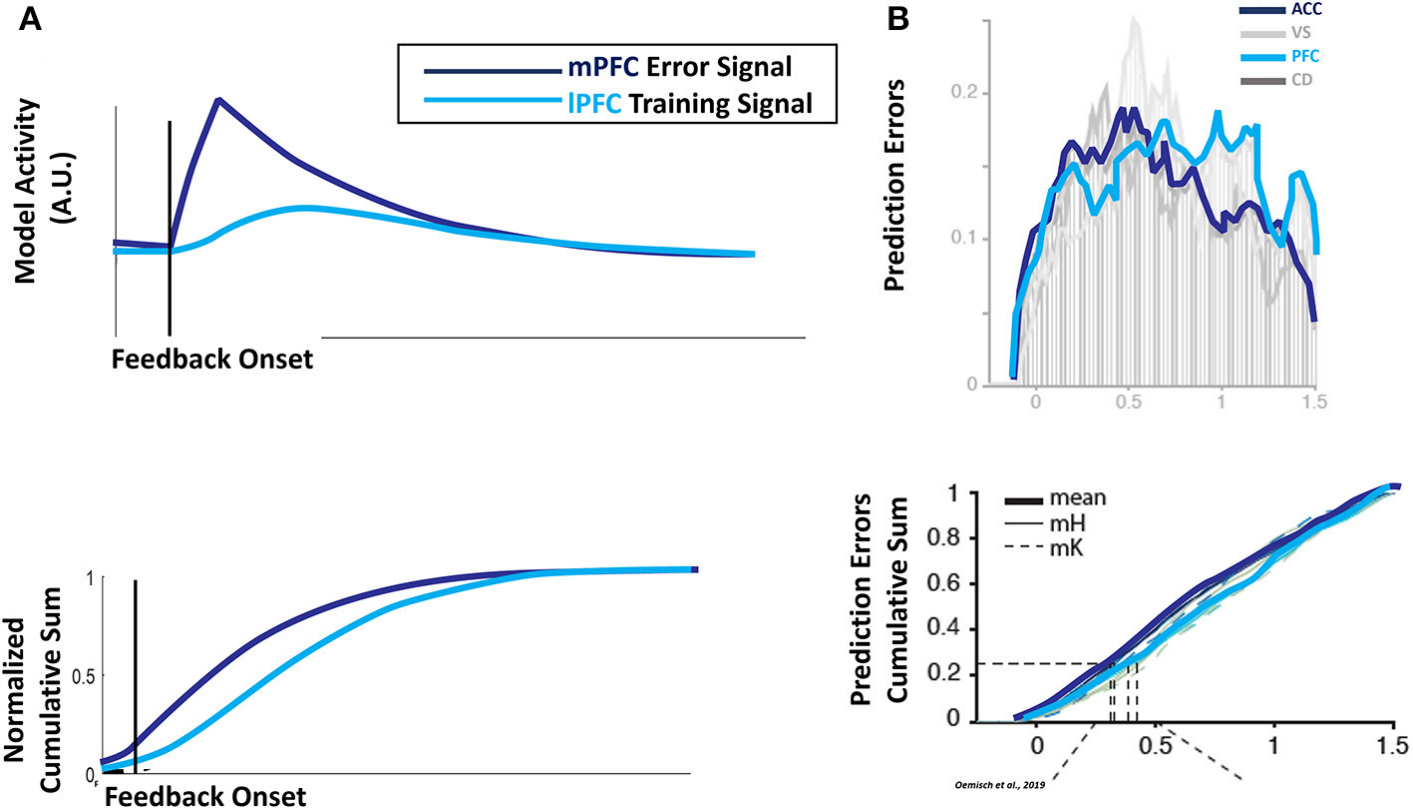
Temporal dynamics of error and prediction. **(A)** Performance-related error signals in the model mPFC at the lowest hierarchical level evolve rapidly following the onset of feedback, peaking soon after the onset of task feedback (**top panel, dark blue line)** and tailing off rapidly (**bottom panel, dark blue line)**. Proxy outcomes used to train superior hierarchical levels in lPFC are a composite of error signals calculated by inferior levels and the contents of WM, with a consequent lag in the temporal profile (**top panel, light blue line)** and lingering activity (**bottom panel, light blue line**). **(B)** The relative temporal profiles of error and composite training signals matches reward predictions errors observed in monkey mPFC and lPFC performing the reversal task, with spiking activity for prediction error units peaking in mPFC/ACC prior to lPFC (**top frame**) and prolonged activity in lPFC relative to ACC/mPFC (**bottom frame**). Figures adapted with permission from Oemisch et al. (2019).

Naturally, since the proxy outcome depends on the lower-level error signal, the evolution of error units in mPFC in our simulations precedes the development of activity in lPFC training units (Figure 5A, top panel): the mPFC error signal ramps up rapidly at the onset of feedback, and decays quickly following feedback offset. In contrast, the lPFC training signal lags the mPFC error signal, and its activity is temporally blurred. Although the relative onset of the error and training signals is prefigured by the architecture of the HER model, the relative distribution (Figure 5A, bottom panel) of the signals emerges only due to the temporal dynamics introduced in these simulations. This emergent pattern qualitatively matches data recorded from monkey dACC and lPFC during performance of the reversal task (Figure 5B; Oemisch et al., 2019), and adds to the already considerable array of effects the HER model has been applied to (Alexander and Brown, 2015, 2018). Furthermore, the HER model provides a mechanistic explanation for both why prediction errors are observed in multiple brain regions during feedback processing, and for their relative time courses. Instead of multiple areas of the brain engaging in independent calculation of prediction errors, the HER model proposes that a single outcome prediction error calculation propagates through the cognitive control network in a bottom-up fashion.

### Information Flow

So far, our results are suggestive of how mPFC and lPFC interact during preparatory and feedback periods of a trial. To determine how these mPFC/lPFC interactions develop during a trial we turn to Granger causality (Luo et al., 2013) as a measure of how well one variable can be predicted by lagged values of another variable. Here, the variables are the unit activities in mPFC and lPFC and Granger causality indexes whether unit activity in one area is better predicted by the preceding unit activity from the other area than by its own past. Granger causality was computed for a time lag of 50 model cycles for both error-related and prediction units in the model, and trial timing was standardized—even if a response was indicated prior, the model was simulated for 300 cycles following the onset of a stimulus.

**Figure 6:**
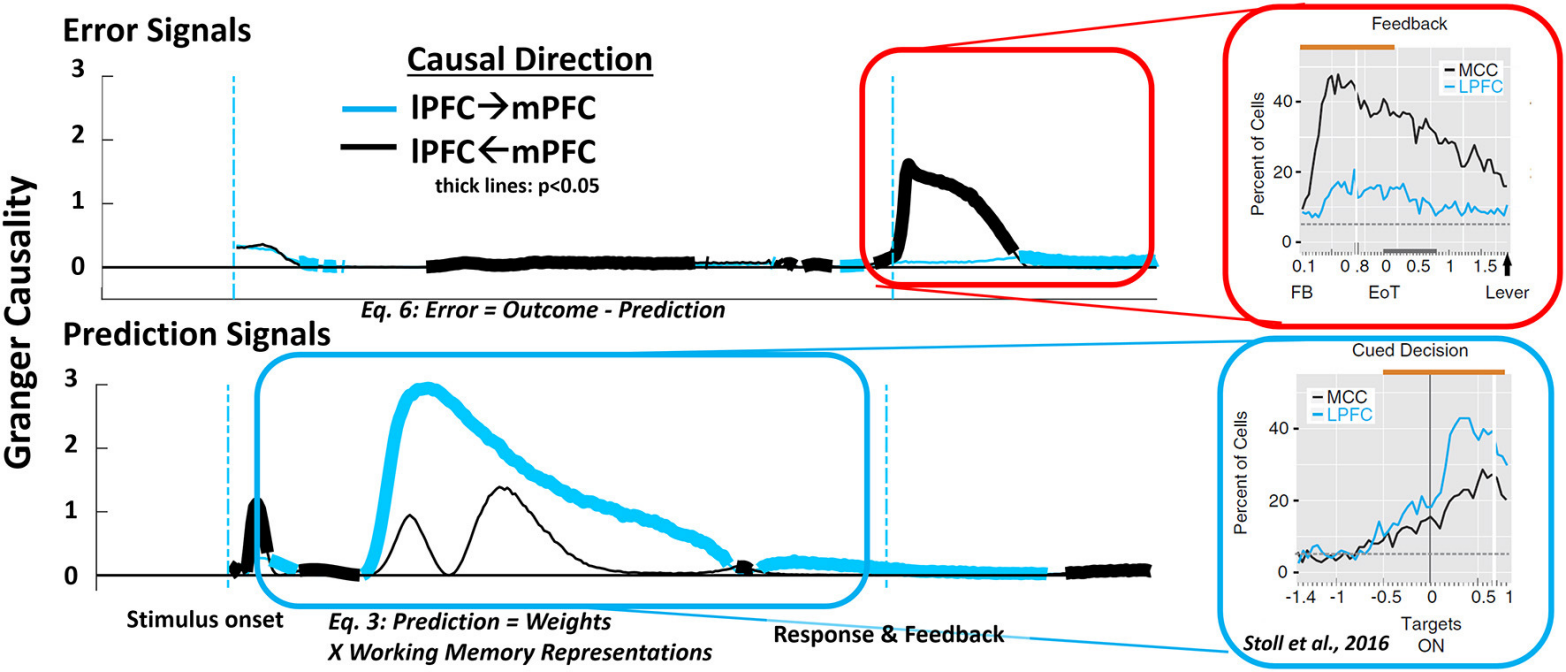
Analysis of causality in the model shows a transient causal relationship of mPFC units to lPFC units following behaviorally salient events such as feedback (**top row**). This causal relationship is reversed during trial periods involving the preparation and execution of responses (**bottom row**). The dynamic shift in causal direction over the course of a trial matches similar patterns observed in monkey mPFC and lPFC during feedback processing and cued performance [right frames, adapted with permission from Stoll et al. (2016)].

We first tested for causally significant error signals (Equation 6) and found they emerged prominently following performance-related feedback (Figure 6, top row). Immediately following the onset of feedback, Granger causality for the influence of mPFC on lPFC was significant at a level of *p* < 0.05 (two sample *t*-test, df = 19, Bonferroni corrected), and remained so until feedback-related activity naturally decayed. lPFC also causally influenced mPFC during the feedback epoch of the task at a significance level of *p* < 0.05 (two sample *t*-test, df = 19, Bonferroni corrected), but only after an outcome feedback had ended.

We next analyzed Granger causal effect of the prediction signal of the model (Figure 6, bottom row). We found that prediction signals at the mPFC representation of the model immediately following stimulus onset Granger-cause activity in lPFC (*p* < 0.05; two sample *t*-test, df = 19, Bonferroni corrected). While the HER model contains no mechanism by which predictions in mPFC influence processing activity in lPFC, Granger causality only indicates whether one signal can be predicted by previous values of another signal. In this case, predictive signals in mPFC and lPFC are correlated, but mPFC signals develop more rapidly than lPFC signals, producing a significant Granger causality effect. Following this transient effect, Granger causality for the influence of lPFC on mPFC becomes significant during the delay period prior to the generation of a response (Figure 6, bottom frame). This finding is consistent with the role of lPFC in maintaining information and implementing control demands. These simulation results are consistent with monkey neurophysiological studies demonstrating differences in the relative onset and peak activity of neurons in mPFC and lPFC that depend on the trial epoch (Figure 6, right panels; Stoll et al., 2016).

## Discussion

In this manuscript, we have described additional simulations of the HER model in which real-time temporal dynamics were introduced to the model. The results of these simulations provide additional perspective on how the activity of mPFC and lPFC, as components of a hierarchical predictive coding framework (Alexander and Brown, 2018), might develop and interact following salient task events, and how the relative direction of this interaction evolves over the course of a trial. Beyond simply exploring the dynamics of model activity, our simulations demonstrate how the HER model can further account for additional single-unit (Oemisch et al., 2019), behavioral (Wylie and Allport, 2000), and network effects (Stoll et al., 2016) previously reported in the literature.

At the level of neurons, the activity of single units in the HER model corresponding both to lPFC and mPFC, is observed to ramp-up following the onset of a task-relevant stimulus (Figure 3). Previous real-time models of mPFC (Alexander and Brown, 2011, 2014) have likewise shown units with ramping activity profiles, similar to those of reward- and error-predicting neurons in monkey mPFC (Amador et al., 2000; Amiez et al., 2006). The HER model, conceived as a temporally-coarse hierarchical extension to the PRO model (Alexander and Brown, 2015), was unable to replicate this pattern; by re-introducing real-time dynamics, and consequently reproducing effects from the PRO model that depended on temporal processes, our simulations underscore that the principal role and computational mechanisms attributed to mPFC by the PRO model remain intact in the HER model.

Furthermore, the simulations in this manuscript extend real-time processing to the registration of feedback and the development of error signals. The original PRO model (Alexander and Brown, 2011) was developed using temporal difference (TD) learning formulations (Sutton, 1988) in which the temporal profile of feedback signals (and subsequent error signals) was specified by the modeler (e.g., either as a punctate event or a box car profile). Here, the duration and magnitude of feedback signals is still modeler-defined, but the development of activity registering these signals is described by the same timing equations used to model all other unit activity in the model. By extending temporal dynamics to feedback processes, the simulations here are able to capture the temporal profile of error-signaling units recorded from monkey mPFC and lPFC, as well as the relative onset and decay of these signals (Figure 5).

Specifically, simulated error-related signals in mPFC peak earlier and decay more rapidly than signals observed in lPFC, consistent with recent reports (Shen et al., 2015; Oemisch et al., 2019). The HER model explains this through the role of mPFC in training error representations in lPFC: error signals generated directly by outcome feedback in the model are combined with active representations of task-stimuli to derive higher-order outcome and error signals (Equation 8), represented in lPFC. As the development of these higher-order signals is mechanically subsequent to direct error signals (Figure 1), the dynamics specified in Equation (11) dictate a later peak and lingering activity. Furthermore, the “proxy” outcome signals derived in lPFC are required for subsequent higher-order error calculations, suggested by the HER model to be carried out in hierarchically-superior regions of mPFC (Figure 4), and these error signals are subject to additional lag as lower-order error and training signal computations that support their calculation develop. The HER model thus provides a mechanistic explanation for the relative time course of error signals in caudal-to-rostral regions of mPFC (Polli et al., 2005).

Although computation of error(Equation 6) and prediction signals (Equation 3) is ongoing throughout our causality analysis over the entire course of a trial reveals that the net direction of information flow depends both on the type of information (prediction or error) computed, as well as the period within a trial. Following salient task events, such as stimulus onset or delivery of feedback, information in the model flows primarily from mPFC to lPFC, while during periods in which a salient event is expected but has yet to occur, information flows principally from lPFC to mPFC. This pattern maps well both to functional roles attributed to these regions, as well as the observed time course of interactions. Functionally, mPFC has long been associated with processing novel or behaviorally-relevant events, especially the occurrence of errors (Gehring et al., 1990) or otherwise surprising (Jessup et al., 2010; Ide et al., 2013) stimuli, while lPFC is implicated in slower processes involving information maintenance (Sawaguchi and Goldman-Rakic, 1991), representing task structure (Badre and D'Esposito, 2007), or implementing control in preparation for upcoming demands (Botvinick et al., 2001). This temporal dissociation, implied in the architecture of the HER model (Alexander and Brown, 2015) is made explicit in this manuscript, and the relative timing and flow of information in the model is consistent with human and monkey studies of PFC (Taren et al., 2011; Shen et al., 2015; Stoll et al., 2016; Oemisch et al., 2019).

The model distinguishes different levels of a computational hierarchy, which we associate with different brain areas to highlight the overarching functional contributions of a specific brain area. The association of the first (lowest) level of the hierarchy with mPFC and the next higher hierarchical level with lPFC should not imply that these areas are exclusively representing information pertaining to these hierarchical levels. Rather, task related information about the value of stimulus features and the type of prediction errors can be decoded from model units at all levels, albeit to different degrees. This observation is consistent with a large body of literature demonstrating that prefrontal cortical neurons code task variables with mixed selectivity (Fusi et al., 2016), whereby individual neurons encode multiple task variables, i.e., they mix information about task rules, feature values, and outcome variables in their firing independent of the specific source of the information (Bernardi et al., 2020). According to these insights, mPFC and lPFC will host neurons tuned to similar task variables, but will show a “representational gradient” (Kyriazi et al., 2020) showing more prominent encoding of feedback information in mPFC and a more abstract stimulus value code in lPFC (Rigotti et al., 2013; Bernardi et al., 2020). Our model depiction is consistent with these insights and specifies the actual computations that might give rise to these representational gradients.

Finally, by introducing temporal dynamics, we were able to use the HER model to replicate sequential trial effects that are a staple of the cognitive control literature (Wylie and Allport, 2000; Fecteau and Munoz, 2003). Unsurprisingly, reaction times for the model are the most rapid for trials in which all features of the chosen stimulus are identical, and slowest for trials in which all features have changed relative to the previous trial. Of interest, however, are trials in which only one relevant feature (out of two in the current study) changes. In these cases, the identity of the changing stimulus can have differential effects on reaction time. The HER model solves structured tasks by decomposing stimulus dimensions hierarchically (Alexander and Brown, 2015): dimensions that are “concrete,” i.e., those that most directly inform the eventual response, are preferentially encoded at the lowest hierarchical level, while more abstract features are encoded at superior hierarchical levels. The simulations reported here suggest that changes in the concrete decision variable (in this case, the direction of the target response) may have a more profound influence on reaction time than changes in the more abstract variable. Recent work (Vassena et al., 2019) has begun to explore how interfering with the structure of a task through manipulations of presentation order might influence decision making and performance. The results of the present study suggest a complementary approach in which the differences in performance elicited through feature changes might be used to infer the representation of task structure.

In summary, the simulations of the extended HER model reported in this manuscript demonstrate that including temporal dynamics endows the model with additional explanatory power, and provides the basis for additional work investigating the function and interaction of regions within PFC, as well as how they contribute to behavior. More generally, the HER model, as an instance of predictive coding, suggests how additional regions in PFC may be organized (Alexander et al., 2017); specifically, the HER model is primarily concerned with how information is integrated in order to generate responses, but may have little to say about how information is acquired to begin with. It is possible that additional regions implicated in cognitive control may be integrated with the HER framework to describe information is actively selected and interpreted to assist in adaptive behavior.

## Data Availability Statement

The original contributions presented in the study are included in the article/supplementary material, further inquiries can be directed to the corresponding author/s.

## Author Contributions

WA and TW conceived of the study and wrote the manuscript. WA conducted computational simulations. All authors contributed to the article and approved the submitted version.

## Conflict of Interest

The authors declare that the research was conducted in the absence of any commercial or financial relationships that could be construed as a potential conflict of interest.
